# Utilizing chitooligosaccharides from shrimp waste biodegradation via recombinant chitinase A: a promising approach for emulsifying hydrocarbon and bioremediation

**DOI:** 10.1186/s12934-024-02388-z

**Published:** 2024-05-02

**Authors:** Shaimaa A. Nour, Maha T. H. Emam, Ghada M. El-Sayed, Ebtehag A. E. Sakr

**Affiliations:** 1 Chemistry of Natural and Microbial Products Department, Pharmaceutical and Drug Industries Research Institute, National Research Centre (NRC), 33 El-Behouth St., Giza, 12622 Dokki Egypt; 2 Genetics and Cytology Department, Biotechnology Research Institute, National Research Centre, Giza, Dokki Egypt; 3 Microbial Genetics Department, Biotechnology Research Institute, National Research Centre, Giza, Dokki Egypt; 4 Botany Department, Faculty of Women for Arts, Science and Education, Ain Shams University, Cairo, Egypt

**Keywords:** Hydrocarbon removal, Bioemulsifier, Shrimp waste, Chitinase A, Chitooligosaccharides, Gene expression

## Abstract

**Background:**

Hydrocarbon pollution stemming from petrochemical activities is a significant global environmental concern. Bioremediation, employing microbial chitinase-based bioproducts to detoxify or remove contaminants, presents an intriguing solution for addressing hydrocarbon pollution. Chitooligosaccharides, a product of chitin degradation by chitinase enzymes, emerge as key components in this process. Utilizing chitinaceous wastes as a cost-effective substrate, microbial chitinase can be harnessed to produce Chitooligosaccharides. This investigation explores two strategies to enhance chitinase productivity, firstly, statistical optimization by the Plackett Burman design approach to  evaluating the influence of individual physical and chemical parameters on chitinase production, Followed by  response surface methodology (RSM) which delvs  into the interactions among these factors to optimize chitinase production. Second, to further boost chitinase production, we employed heterologous expression of the chitinase-encoding gene in *E. coli* BL21(DE3) using a suitable vector. Enhancing chitinase activity not only boosts productivity but also augments the production of Chitooligosaccharides, which are found to be used as emulsifiers.

**Results:**

In this study, we focused on optimizing the production of chitinase A from *S. marcescens* using the Plackett Burman design and response surface methods. This approach led to achieving a maximum activity of 78.65 U/mL. Subsequently, we cloned and expressed the gene responsible for chitinase A in *E. coli* BL21(DE3). The gene sequence, named *SmChiA*, spans 1692 base pairs, encoding 563 amino acids with a molecular weight of approximately 58 kDa. This sequence has been deposited in the NCBI GenBank under the accession number "OR643436". The purified recombinant chitinase exhibited a remarkable activity of 228.085 U/mL, with optimal conditions at a pH of 5.5 and a temperature of 65 °C. This activity was 2.9 times higher than that of the optimized enzyme. We then employed the recombinant chitinase A to effectively hydrolyze shrimp waste, yielding chitooligosaccharides (COS) at a rate of 33% of the substrate. The structure of the COS was confirmed through NMR and mass spectrometry analyses. Moreover, the COS demonstrated its utility by forming stable emulsions with various hydrocarbons. Its emulsification index remained stable across a wide range of salinity, pH, and temperature conditions. We further observed that the COS facilitated the recovery of motor oil, burned motor oil, and aniline from polluted sand. Gravimetric assessment of residual hydrocarbons showed a correlation with FTIR analyses, indicating the efficacy of COS in remediation efforts.

**Conclusions:**

The recombinant chitinase holds significant promise for the biological conversion of chitinaceous wastes into chitooligosaccharides (COS), which proved its potential in bioremediation efforts targeting hydrocarbon-contaminated sand.

**Supplementary Information:**

The online version contains supplementary material available at 10.1186/s12934-024-02388-z.

## Introduction

Many dangerous substances, such as n-alkane, cycloalkane, and aromatic hydrocarbons, are found in petroleum-based products. Contaminated sites may result from operational activities and accidents during large-scale petroleum exploration, transport, processing, storage, and consumption [1]. Because of its toxicity, hydrocarbon pollution is still a major environmental issue. It is recognized that hydrocarbon components are neurotoxic organic pollutants and carcinogens [2]. Because of this, issues over the removal of hydrocarbons from the environment are crucial. In addition to being exceedingly expensive, the physical and chemical methods of removing petroleum are now irrelevant. Because it is quite inexpensive and will result in full mineralization, bioremediation is regarded as the most promising approach for the treatment of these contaminated sites [2]. The use of bioproducts derived from microbial enzymes as a bioemulsifier for hydrocarbon cleanup is thought to be an inventive, economical, and environmentally friendly method.

Because they have advantages over synthetic emulsifiers, bioemulsifiers have gained popularity in a variety of industrial and environmental applications [3]. For possible use in the petroleum industry, bioemulsifiers have been thoroughly studied [4]. According to Pal et al. [5], nature has provided an abundance of materials for resource restoration, with chitin being one of the most significant and widely accessible biomaterials worldwide.

*N* acetyl-d-glucosamine (GlcNAc) units connected by β-1,4 linkages make up the nitrogenous polysaccharide chitin [6]. It is an insoluble, highly resistant to degradation polymer that comes in second place to cellulose [7]. Moreover, the sustainable valuation of chitinous waste is vital in averting environmental degradation due to its excessive generation. There hasn’t been much use of chitinous biowaste to produce chitin and chitooligosaccharides thus far. Within this framework, chitin-active enzymes serve as essential instruments for managing chitin waste while concurrently producing products with additional value [8]. Enzymes called chitinases break down the β-1,4-glycosidic bonds found in chitin. In industrial applications, shellfish waste that contains chitin is broken down enzymatically to produce chitooligosaccharides (COS), which can be used to make high-value products. Nevertheless, the industrial value of chitinases is limited due to their low tolerance and low efficiency of production. Finding chitinases with increased enzymatic activity and tolerance is therefore crucial [9].

In addition, the physicochemical environment must be optimized for the industrial production of any biological product before it can be scaled up and commercialized. One of the most effective statistical tools for creating models, planning experiments, assessing the impacts of different variables, and locating the ideal circumstances for a multifactorial fermentation process is response surface methodology (RSM) [10]. Compared to univariate solutions, the methodology is more exact, practical, and industrially acceptable from a techno-economic standpoint [11, 12]. Additionally, compared to native microorganisms, the development of recombinant enzymes in an appropriate host can offer a far higher yield and a purer product with less processing time [13].

Chitinases play diverse physiological roles and are found across various species, spanning bacteria, viruses, fungi, insects, plants, and animals. Among these, Serratia stands out as a particularly efficient bacterial genus known for chitinase production [14]. Within *S. marcescens*, the enzymes ChiA, ChiB, ChiC, ChiD, and CBP21 play key roles in chitin breakdown [15–17]. Both ChiA and ChiB are exochitinases but ChiA is  the most potent enzyme against insoluble chitins [18, 19]. To our knowledge, this is the first instance of utilizing COS derived from the biodegradation of shrimp powder, serving as the chitin source via recombinant chitinase A for bioemulsification and hydrocarbon removal. In the present study, the production of chitinase A from *S. marcescens* strain NRC408 was enhanced using two different approaches. First, optimization of chitinase A production from *S. marcescens* via Plackett–Burman and RSM designs. Second, cloning and heterologous expression of recombinant enzyme in *E. coli.* Then biochemical chaion of chitinase A was done to provide effective utilization of this enzyme for the degradation of chitin waste. Furthermore, the hydrolytic efficiency of chitinase A was evaluated for the biological production of COS. The identification of the synthesized COS was determined using advanced analytical techniques. The produced COS was tested for emulsification, and stability under different conditions of temperature, pH, and salinity, and was also checked for its effect on removing hydrocarbons.

## Results

### Statistical optimization of chitinase A production from *S. marcescens*

**Fig. 1:**
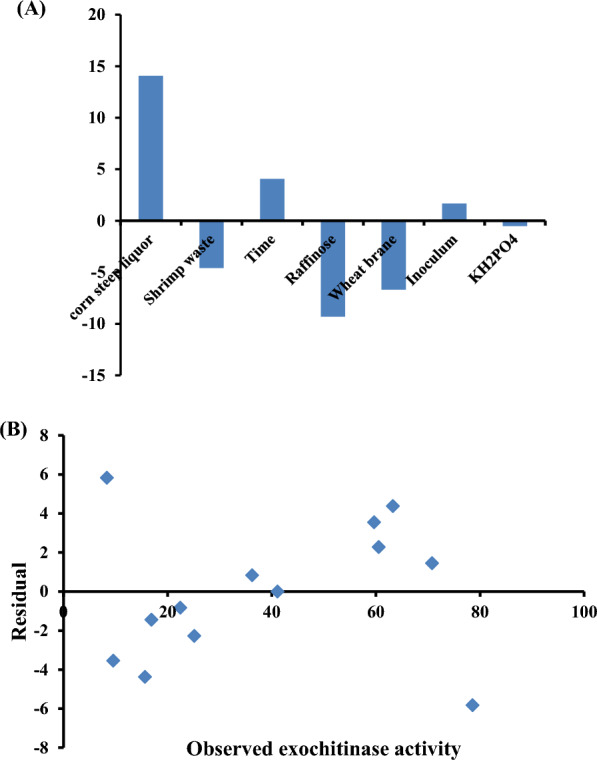
**A** Main effects of independent variables on *S. marcescens* 408 chitinase A production according to the results of the PBD. **B** Residual plot of the observed-predicted values (residuals) versus the response (optimization process) of chitinase A activity
  First-order model Eq. (1) could be used to illustrate the relationship between independent factors and SmChiA production:
  1 $${\text{Y}}_{{{\text{Activity}}}} = {32}.{63584} + {7}.0{\text{29172X}}_{{1}} - {2}.{\text{29298X}}_{{2}} + {2}.0{32}0{\text{55X}}_{{3}} - { 46}.{\text{4922X}}_{{4}} - { 13}.{\text{3784X}}_{{5}} - { 2}.{\text{59X}}_{{6}} - {2}.{\text{31X}}_{{7}}.$$
B. Central composite design (CCD)
  To enhance and optimize the chitinase A produced by *S. marcescens*, statistical and mathematical analysis of multivariable data obtained from RSM of CCD was crucial. The key point of the CCD was the medium consisting of the following (g/L): steep liquor (25); shrimp waste (20); corn raffinose (1); wheat bran (5); KH_2_PO_4_ (15) and inoculated with 4% of inoculum medium and incubated at 30 °C under shaking (180 rpm) for 24 h. Table 2 shows the 13 trials matrix of CCD with 3 components (corn steep liquor, raffinose, and wheat brane) derived from PBD and three levels (-1, 0, and + 1) containing 6 repetitions at the central point. Multiple regression analysis of the observed data produced a second-order polynomial equation, Eq. (2), whose mathematical optimal point was ascertained as follows:
  2 $$Y_{Activity} = - {33}.{13} - 11.098X1 + 127.48X2 + 92.94X3 + 7.66X12 \, - 151X22 \, - X32 - 67.3X1X2 + \, 31.1X1X3 + \, 923.5X2X3.$$
  where response (*Y*_*Activity*_) was predicted chitinase A production and (X1, X2, and X3) were the codes of most effective variables corn steep liquor, raffinose, and wheat brane, respectively.

A. Using Plackett–Burman designs (PBD)
  PBD was used to identify the factors primarily influencing chitinase A production among a wide range of culture conditions and medium composition. Table 1A displays nine combination matrices containing seven independent factors for the manufacture of chitinase A. The highest activity was obtained by the combination number 2 which increased the enzyme activity from 11.58 to 41.12 U/mL. The main effect of the variables revealed that shrimp waste, raffinose, wheat bran, and KH_2_PO_4_ had a negative main effect, whereas corn steep liquor, time, and inoculum had a positive main effect (Fig. 1A). The importance of each coefficient could be ascertained using the P-value, which also helped to identify the pattern of the interactions between the variables. The statistical examination of the data revealed that terms with smaller P-values (P < 0.05) had asignificant effect on Chitinase A production. Table 1B displays the t-test, p-value, and regression coefficients for seven independent variables. The results show that the P-values for corn steep liquor, raffinose, and wheat bran are 0.002274, 0.007504, and 0.018737, respectively. These findings showed that the most important factors influencing the production of chitinase A were raffinose, wheat bran, and corn steep liquor. When the R^2^ value was more than 0.9, the correctness of the model was verified. Therefore, the applied model proved to be acceptable in this study, with an even higher R^2^ value of 0.971, suggesting that the variables under investigation produced a variance of 97.1% in enzyme activity.

**Table 1:** Placket-Burman design for evaluating the factors influencing *S. marcescens* 408 chitinase A production (A) and its statistical analysis (B)

(A) Trial	Corn steep liquor (g/L)	Shrimp waste (g/L)	Time	Raffinose (g/L)	Wheat bran (g/L)	Inoculum	KH_2_PO_4_ (g/L)	U/mL
1	5 (−)	20 (−)	1 (−)	3 (+)	15 (+)	4 (+)	5 (−)	11.58 ± 1.315
2	25 (+)	20 (−)	1 (−)	1 (−)	5 (−)	4 (+)	15 (+)	41.12 ± 2.89
3	5 (−)	60 (+)	1 (−)	1 (−)	15 (+)	2 (−)	15 (+)	14.11 ± 1.336
4	25 (+)	60 (+)	1 (−)	3 (+)	5 (−)	2 (−)	5 (−)	26.06 ± 1.279
5	5 (−)	20 (−)	3 (+)	3 (+)	5 (−)	2 (−)	15 (+)	20.15 ± 0.820
6	25 (+)	20 (−)	3 (+)	1 (−)	15 (+)	2 (−)	5 (−)	37.32 ± 1.336
7	5 (−)	60 (+)	3 (+)	1 (−)	5 (−)	4 (+)	5 (−)	27.04 ± 0.989
8	25 (+)	60 (+)	3 (+)	3 (+)	15 (+)	4 (+)	15 (+)	24.61 ± 1.909
9	15 (0)	40 (0)	2 (0)	2 (0)	10 (0)	5 (0)	10 (0)	22.08 ± 0.530
10	15 (0)	40 (0)	2 (0)	2 (0)	10 (0)	5 (0)	10 (0)	22.08 ± 0.530
11	15 (0)	40 (0)	2 (0)	2 (0)	10 (0)	5 (0)	10 (0)	22.08 ± 0.530

(B)	Coefficients	Standard error	t Stat	P-value
Intercept	32.63584	3.57004	9.141588	0.002767
Corn steep liquor	7.029172	0.718985	9.776515	0.002274
Shrimp waste	− 2.29298	0.718985	− 3.18919	0.049742
Time	2.032055	0.718985	2.826282	0.066393
Raffinose	− 46.4922	7.189854	− 6.46636	0.007504
Wheat bran	− 13.3784	2.875942	− 4.65182	0.018737
Inoculum	− 0.36306	0.189709	− 1.91379	0.15156
KH_2_PO_4_	− 0.50604	1.437971	− 0.35191	0.748173
The model’s summary				
Multiple R, 0.985				
R^2^, 0.971				
Adjusted R^2^, 0.904				
Standard error, 2.704

**Table 2:** Examined concentration of the key variables and results of the CCD experiment

Runs	Corn steep liquor	Raffinose	Wheat bran	Activity (U/mL)	Predicted (U/mL)
1	20 (−)	0.5 (−)	5 (0)	15.66 ± 0.537	20.035
2	30 (+)	0.5 (−)	5 (0)	60.55 ± 2.283	58.27
3	20 (−)	1.5 (+)	5 (0)	25.11 ± 2.276	27.39
4	30 (+)	1.5 (+)	5 (0)	63.27 ± 2.078	58.895
5	20 (−)	1 (0)	3 (−)	8.33 ± 0.855	2.505
6	30 (+)	1 (0)	3 (−)	22.44 ± 1.329	23.27
7	2 (−)	1 (0)	7 (+)	36.24 ± 0.77	35.41
8	30 (+)	1 (0)	7 (+)	78.56 ± 0.692	84.385
9	25 (0)	0.5 (−)	3 (−)	16.89 ± 0.791	18.34
10	25 (0)	1.5 (+)	3 (−)	9.55 ± 0.608	13.095
11	25 (0)	0.5 (−)	7 (+)	59.66 ± 1.654	56.115
12	25 (0)	1.5 (+)	7 (+)	70.79 ± 1.739	69.34
13	25 (0)	1 (0)	5 (0)	41.11 ± 1.0182	41.11

R^2^, 0.980037; adjusted R^2^, 0.920149; *P*-value, 0.020988; F,16.36446
  Trial number 8 exhibited the highest level of activity, yielding 78.56 U/mL (3.56-fold from baseline medium) at 30 g/L of corn steep liquor, 1 g/L of raffinose, and 7 g/L of wheat bran following a day at 30 °C. Additionally, the response (optimization process) vs. the observed-predicted values (residuals) was plotted in the residual analysis (Fig. 1B), which revealed that the residuals were symmetrically distributed and uniformly distributed across the range, indicating that the average model was accurate for all observed results.
  The prediction of chitinase A production for every trial matrix served as an indication for the proposed model’s validity. Table 2 presents the experimental data, which indicate that the maximal chitinase production observed (78.65 U/mL) was in close agreement with the expected value of 84.385 U/mL. The model’s F- and P-values were determined to be 16.364461 and 0.020988, respectively, and its R^2^ coefficient was found to be 98%, suggesting that the model’s equation could be responsible for the variability in the responses. Therefore, the relationship between the three variables (corn steep liquor, raffinose, and wheat bran) and the chitinase A production may be found using the second-order polynomial equation Eq. (3). The maximum amount of Ex chitinase produced was 78.65 U/mL, which was 3.56 times more than what was produced in the basal medium. Following the precipitation of crude enzyme at ethanol saturation levels between 50 and 60%, 71.44% of the total activity was recovered, resulting in an 8.44-fold increase in specific activity.

### Cloning of *SmChiA* gene in a heterologous expression system

#### Isolation and transformation of *SmChiA* gene into* E. coli* DH5α

**Fig. 2:**
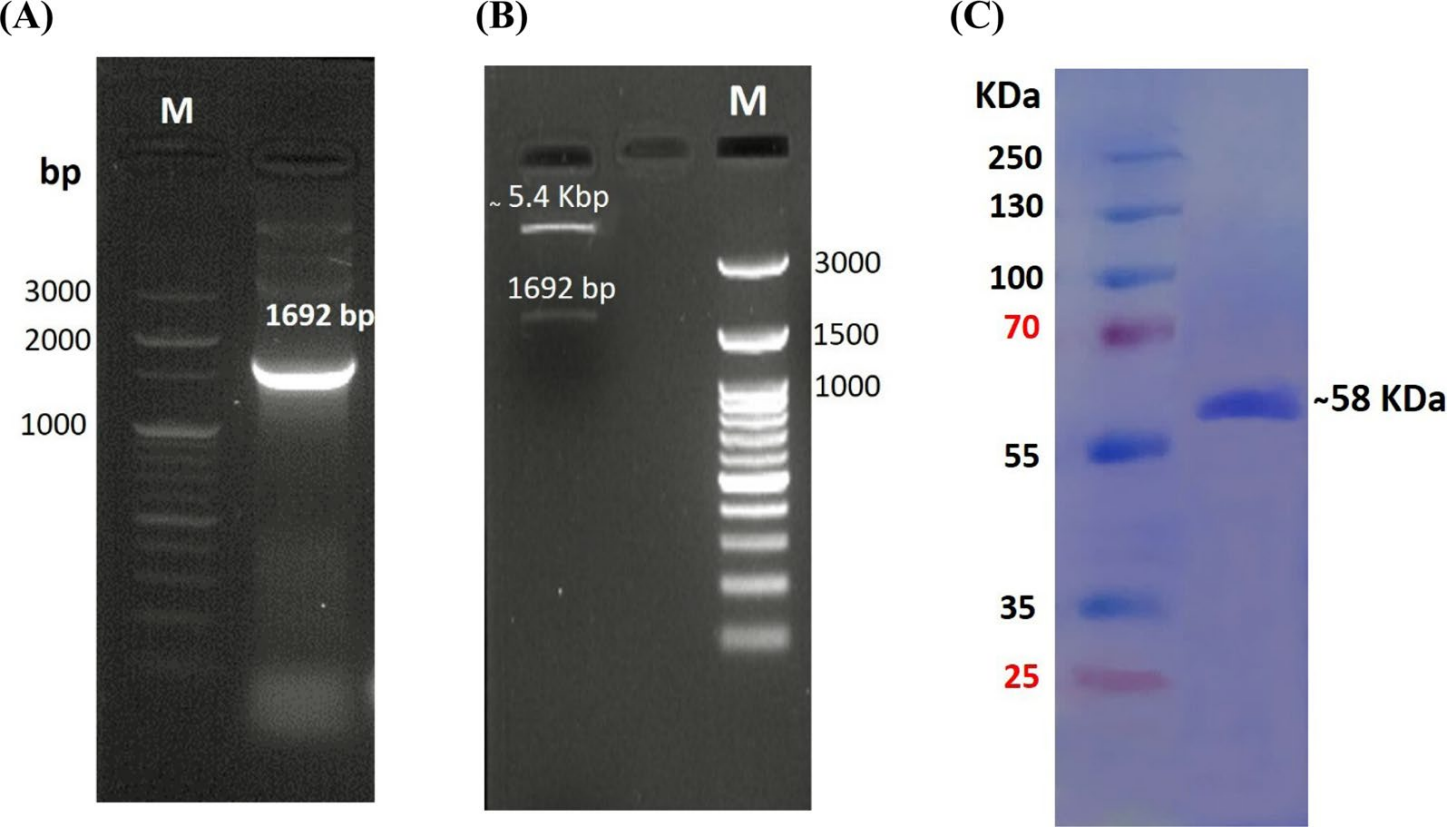
Agarose gel electrophoresis for (**A**) the *SmChiA* gene fragment of strain *S. marcescens* NRC408. **B** Product of double digestion by restriction enzymes (*HindIII* and *EcoRI*) for the constructed SmChiA-pET 28a^+^ plasmid. **C** SDS-PAGE electrophoresis of purified chitinase from transformant *E. coli* BL21 (DE3). Lane M: protein ladder (ThermoFisher)

The chitinase A gene from *S. marcescens* NRC408 was isolated by PCR amplification method using designed specific primers. The isolated gene (named *SmChiA*) was analyzed by agarose gel electrophoresis with 100 bp Plus DNA Ladder (ThermoFisher), the fragment size 1692 pb is shown in Fig. 2A. The purified *SmChiA* gene fragment was first ligated to plasmid pJET1.2/blunt and transformed to *E. coli* DH5α. Then, the *SmChiA* gene fragment was digested by double restriction enzymes (*HindIII* and *EcoRI*), ligated to pET 28a + plasmid, and transformed to *E. coli* DH5α. After that, the plasmid was isolated from the growing clones, and the positive one was confirmed by double restriction enzyme digestion of the constructed SmchiA-pET 28a + plasmid. The digested products were analyzed by agarose gel electrophoresis using a 100 bp DNA ladder (Enzynomics), the result is illustrated in Fig. 2B.

#### Transformation and expression of *SmChiA* gene into* E. coli* BL21

The positive clone was used to isolate the recombinant plasmid SmChiA-pET28a^+^, which was then transformed into the T7 expression host *E. coli* BL21 (DE3). Different IPTG concentrations and incubation temperatures were used to control the expression of the *SmChiA* gene in *E. coli* BL21 (DE3). The optimum temperature was 37 ºC, while the optimum IPTG concentration was 0.2 mM. Figure 2C displays the protein profile of IPTG-induced positive transformants and reveals the presence of a band at 58 kDa that corresponds to the molecular weight of chitinase A. The expression of purified recombinant chitinase has an activity of 228.085 U/mL which exhibits a 2.9-fold increase in activity when compared to statistically optimized chitinase in *S. marcescens* NRC408 strain, so the recombinant chitinase was chosen for the following studies.

#### Sequencing of *SmChiA* gene, phylogeny and chitinase amino acid analysis

**Fig. 3:**
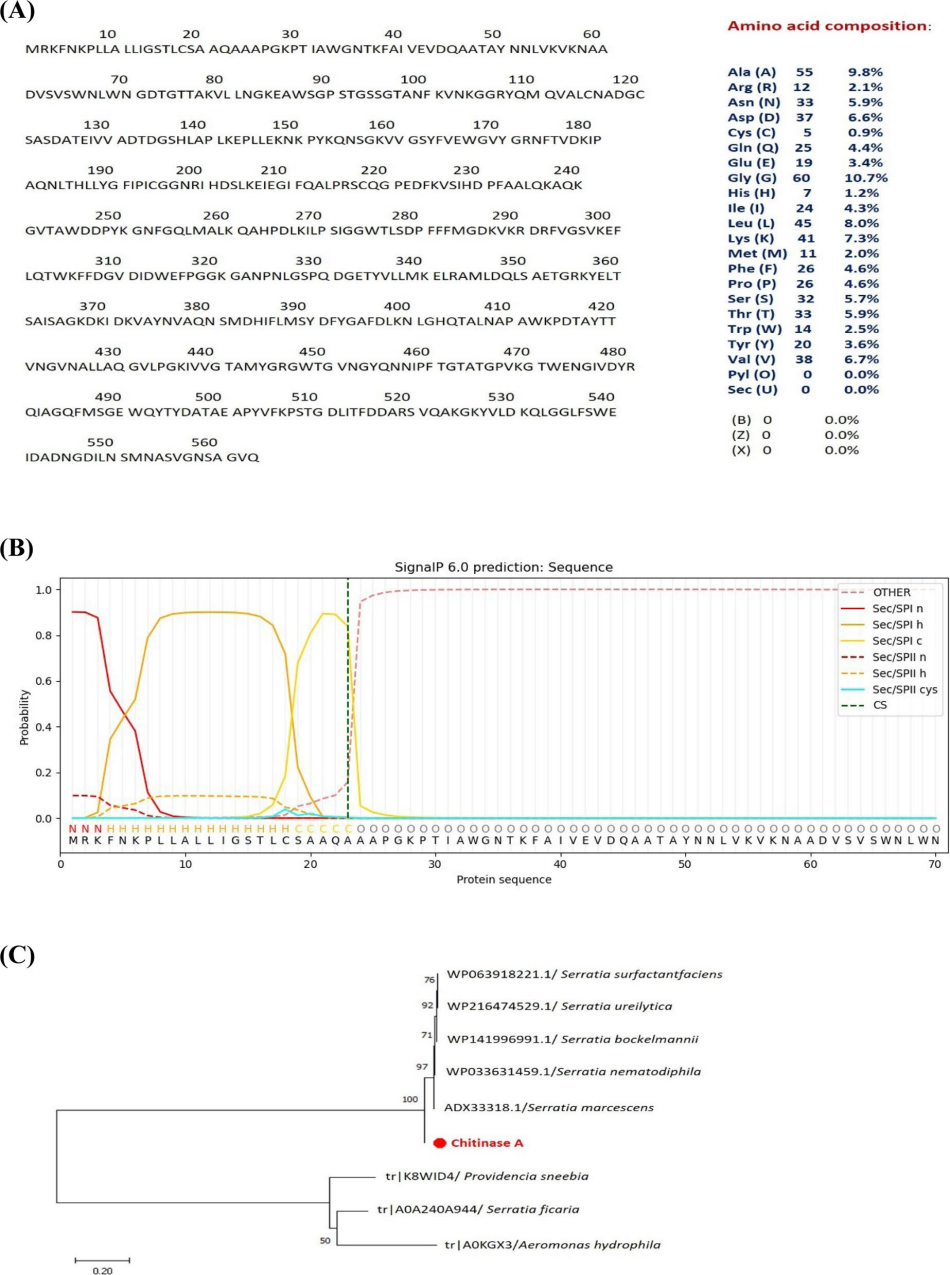
**A** Amino acid composition of chitinase A form *B. S. marcescens* NRC 408 analyzed by Exbasy tool. **B** Signal peptide analysis of chitinase A by Signal P v 6.0. **C** Phylogenetic tree of chitinase A from *S. marcescens* NRC 408 (marked with red color) and closet strains inferred by (MEGA 11)

Vector-specific primers were used to sequence the plasmid (SmchiA-pET28a^+^) in both directions. The sequence was then added to the GenBank database with accession number "OR643436." Using the BlASTn program, the sequence was compared to other sequences in the NCBI database. The outcome demonstrated that the *SmChiA* sequence of *S. marcescens* NRC408 showed significant homology with the other chitinase genes. It shows 99.11% similarity with the chitinase gene of *S. marcescens* (AC: CP013046.2). The open reading frame (ORF) of the *SmChiA* gene is composed of 1692 bp. The analysis of the amino acid composition of chitinase A revealed that the chitinase peptide consists of 563 amino acids, with an estimated molecular weight of approximately 58.56 kDa and a theoretical pI of 5.75. (Fig. 3A). Additionally, the signal IP analysis (Fig. 3B) revealed that chitinase A had a 23-amino acid signal peptide. The phylogenetic relationship between chitinase A from *S. marcescens* NRC408 and its closest strains retrieved from the NCBI database is represented in Fig. 3C**.** The results showed that chitinase A from *S. marcescens* NRC 408 demonstrated strong similarity with *S. marcescens* and *S. nematodiphila*.

### Biochemical characterization of recombinant chitinase A

#### Effects of pH and temperature on the chitinase A activity and stability

**Fig. 4:**
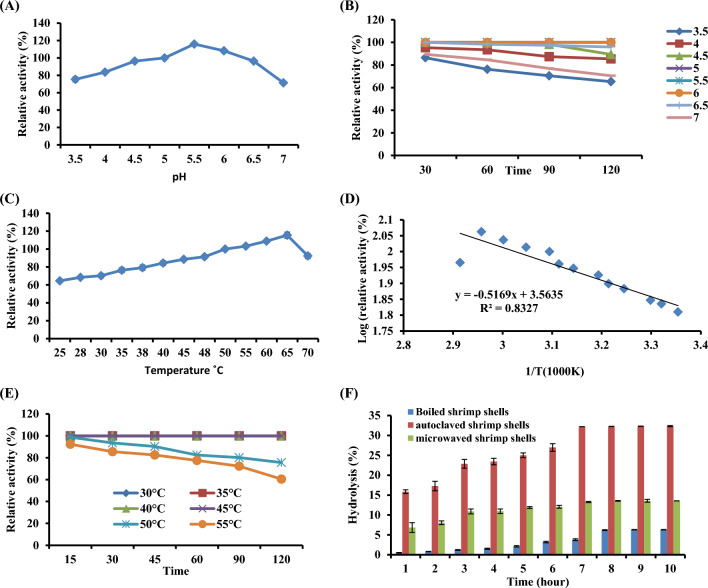
(**A**)The effect of the reaction mixture’s pH on chitinase A activity (**B**) pH stability of chitinase A. **C** Effect of temperature of the reaction on chitinase A. At varied temperatures (**D**) Arrhenius plots to calculate activation energy (Ea) for chitinase A. **E** Temperature-stability profile for chitinase A (**F**) Time course hydrolysis of different treated shrimp by chitinase A

To produce chitooligosaccharides, it is necessary to study the enzyme’s characteristic stability. The effects of pH on chitinase A activity are shown in Fig. 4A. Acetate buffer was used to determine the optimal pH of chitinase A, which was determined to be 5.5. At pH 7, the enzyme’s activity decreased by roughly 29%. The pH stability of the enzyme was displayed in Fig. 4B. The results showed that the enzyme exhibited a broad range of stability at pH 5, 5.5, and 6 for 2 h but the enzyme lost more than 30% of its activity after 2 h at a pH of 3.5. The temperature dependency of chitinase A activities between 25 and 70°C at the optimal pH 5.5 is illustrated in Fig. 4C. We found that 65°C was the ideal temperature for it.

The activation energy and the effectiveness of chemical reactions can be determined using the Arrhenius equation. When a reaction follows Arrhenius’ equation, the log of residual activity is graphed against time to demonstrate a linear relationship that represents the enzyme’s first-order kinetic reaction. The activation energy (Ea) can then be determined using the gradient and intercept. Similar graphs were created by chitinase A, which agreed with the equation that follows:

$${\text{the regression equation was y }} = \, - 0.{\text{5169X }} + { 3}.{5635}.$$

After that, Arrhenius plots were employed to determine the chitinase A catalysis’s activation energy (Ea) (Fig. 4D).

The findings revealed that Ea of the chitinase A was 4.202 kJmol^−^; the lower the value of Ea, the less energy is required to conform to the active site of the enzyme–substrate complex. Chitinase A was therefore thought to be more appropriate for industrial applications due to these features and the fact that it requires a low activation energy value, which will affect the overall cost of industrial processing. Furthermore, as shown in Fig. 4E, the enzyme maintained 75.6% of its activity after 120 min of incubation at 50 °C, while it did not lose any activity after 120 min at 30, 35, 40, or 45 °C. However, after being incubated for 120 min at 55 °C, it lost about 39.56% of its activity.

#### Hydrolysis of shrimp powder by recombinant chitinase and estimation of oligosaccharide level

The hydrolysis mechanism of chitinase A was investigated using shrimp shells as a substrate. chitinase A could quickly hydrolyze autoclaved shrimp shells, as seen in Fig. 4F, releasing a significant amount of reducing sugar GlcNAc as the main outcome (monosugars). The ability of the obtained enzyme to hydrolyze shows that it was an exochitinase. Additionally, the hydrolysis of shrimp shells without treatment produced a low rate of hydrolysis percent output of 6.3% after 10 h, whereas the use of an autoclave produced a greater percentage of around 33% at 7, 8, 9, and 10 h.

### Characterization of chitooligosaccharides

#### ^1^H NMR Spectroscopy

The ^1^H NMR spectra contributed to clarify the chemical structures of COS, and Additional file 1: Fig. S1 displays the attributions of prominent peaks. The presence of GlcNAc residue was confirmed by COS, which displayed a characteristic signal peak at δ 1.92 ppm, which corresponded to the methyl protons of the *N*-acetyl groups.

#### Mass spectrometry

The recombinant chitinase A enzyme produced COS, and the ESI/MS spectra of that COS were obtained in the positive mode (Additional file 1: Fig. S1). Positive ionization mode has been widely used in this approach. [M + H] ^+^ ion peaks make up all of the peaks. Six major peaks were identified, two of which were monomers and the other two were COS. The mass value of 268.12 (Rt; 1.01) for the monomer can be ascribed to *N*,*N*′-diacetyl d-glucosamine. Using m/z values of 410.80, 846.01, 897.89, and 899.93, respectively, retention times (Rt) of 1.23, 6.22, 31.02, and 31.07 min were found for COS (Additional file 1: Fig. S1). As a result, the ESI–MS spectra show that the COS mostly consists of a combination of hetero-oligosaccharides, ranging from monomers to trimers of glucosamine units and N-acetylated glucosamine.

### Evaluation of COS as a bioemulsifier and its application in hydrocarbon removal

#### Emulsification capacity

**Fig. 5:**
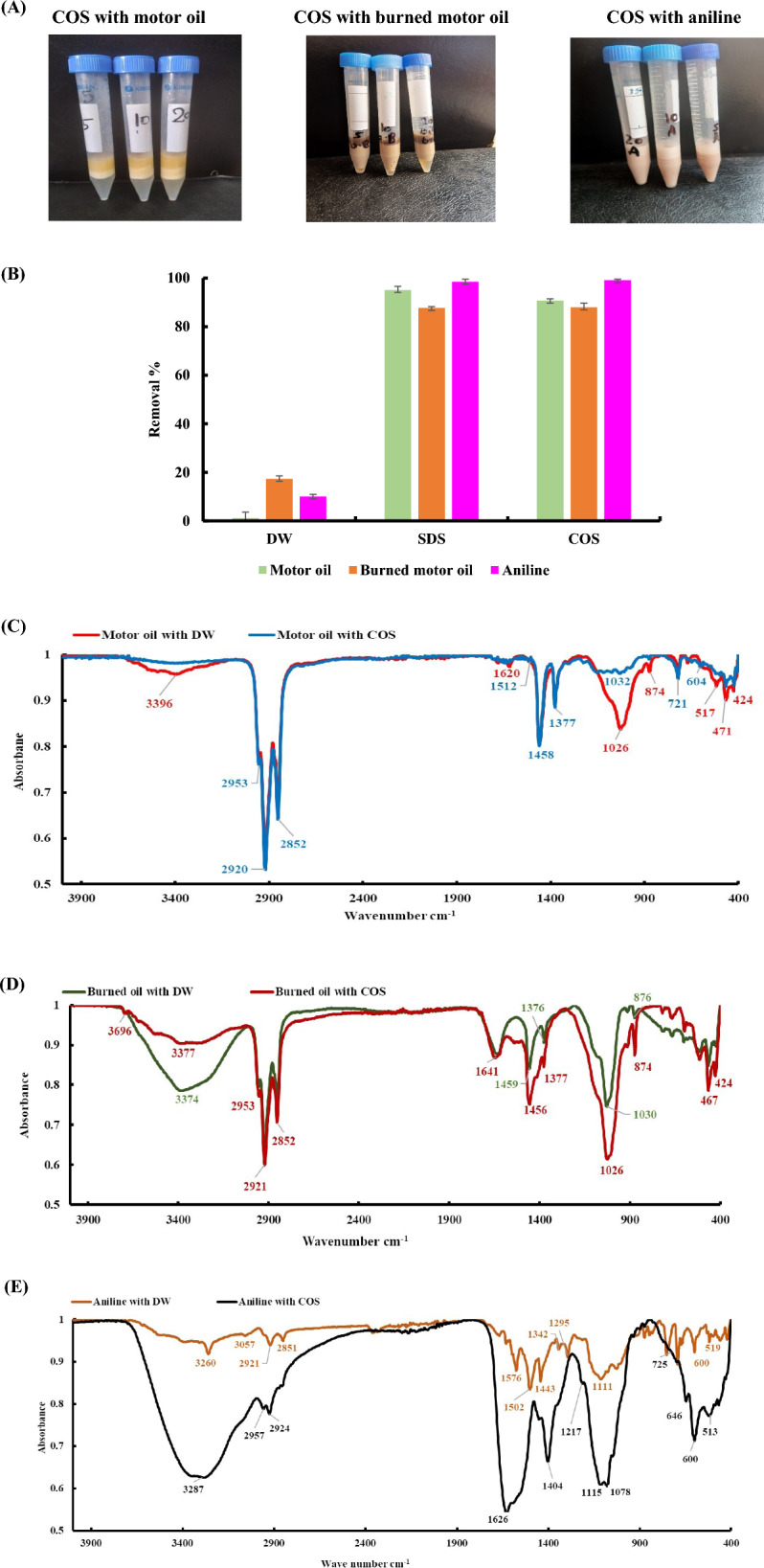
Emulsification activity of different concentrations of COS with motor oil, burned motor oil, aniline (**A**), Removal of hydrocarbons on contaminated sand by distilled water (DW), sodium dodecyl sulfate (SDS), and chitooligosaccharides (COS) (**B**), FTIR spectra of residual motor oil (**C**), burned motor oil (**D**), aniline (**E**) after treatment with COS and distilled water (DW)

The potential of a surface-active substance to convert immiscible liquids into stable emulsions is a useful indicator of that compound’s usefulness. The hydrophobic substrates that the COS generated from *S. marcescens* chitinase evaluated for emulsification are shown in Table 3. The emulsification indices rose in parallel with the COS concentrations. Petroleum ether and cyclohexane were notably poor emulsification substrates. The COS sample showed greatest emulsification activity against motor oil (72.50 ± 2.05%), burned motor oil (86.67 ± 1.17%), and aniline (100 ± 0%) at 20 mg/mL. The outcomes demonstrated that the COS could emulsify several hydrocarbons. Figure 5A shows that motor oil, burned motor oil, and aniline were chosen based on the results.

**Table 3:** Emulsification activity of COS with some oils and hydrocarbons

Oils and hydrocarbons	Emulsification index (%)		
	Different conc. of COS (mg/mL)		
	5	10	20
Vegetable oil	54.17 ± 5.89^c^	60.00 ± 3.54^c^	45.83 ± 3.12^d^
Motor oil	52.50 ± 0^c^	55.83 ± 2.36^c^	72.50 ± 2.05^c^
Burned motor oil	75.00 ± 4.08^b^	80.80 ± 5.14^b^	86.67 ± 1.17^b^
Benzene	25.83 ± 1.17^d^	20.81 ± 3.11^f^	38.33 ± 3.12^e^
Toluene	22.50 ± 2.04^d^	41.67 ± 2.35^d^	40.83 ± 3.11^de^
Xylene	20.83 ± 3.11^d^	30.80 ± 3.12^e^	41.66 ± 3.11^de^
Petroleum ether	5.83 ± 1.18^e^	5.82 ± 1.18^g^	15.00 ± 2.04^g^
Cyclohexane	8.33 ± 1.17^e^	11.67 ± 3.12^g^	30.80 ± 1.19^f^
Aniline	100.00 ± 0^a^	100.00 ± 0^a^	100.00 ± 0^a^
F-value	266.445^***^	226.009^***^	278.903^***^

*COS* chitooligosaccharides

Results are expressed as mean ± standard deviations of values from triplicate experiments

^***^Significant difference at *p* < 0.001

The values in the same column with the same letter are not significantly different

#### COS stability related to emulsification

The efficiency of COS is affected by a number of factors, such as pH, temperature, and salinity. Therefore, while investigating using this metabolite in bioremediation, it’s critical to investigate the impact of these factors. Table 4 displays the COS stability values in relation to temperature, salinity, and pH. As can be observed, at concentrations up to 12% NaCl, the COS preserved more than 62% of the emulsifying activity of the three hydrocarbons (motor oil, burned motor oil, and aniline). These results could be regarded as a good salt resistance of the COS generated by *S. marcescens* chitinase A. COS might be used in saline regions because sea salinity is just 3% worldwide.

**Table 4:** Stability of COS under different environmental factors

	Motor oil	Burned motor oil	Aniline
NaCl (%)			
0	72.50 ± 2.00^b^	80.83 ± 5.14^a^	100.00 ± 0^a^
2	51.67 ± 2.36^d^	72.50 ± 3.53^ab^	100.00 ± 0^a^
4	61.67 ± 4.25^c^	70.00 ± 4.08^ab^	100.00 ± 0^a^
6	88.33 ± 7.72^a^	80.83 ± 4.25^a^	96.66 ± 4.71^ab^
8	61.67 ± 1.18^c^	73.33 ± 5.25^a^	100.00 ± 0^a^
10	61.67 ± 1.17^c^	75.00 ± 0^a^	92.50 ± 4.08^b^
12	62.50 ± 0^c^	62.50 ± 2.04^b^	75.00 ± 2.04^c^
F-value	21.051^***^	3.905^*^	27.581^***^
Temperature (^o^C)			
40	60.83 ± 3.11^ab^	88.33 ± 5.13^a^	100.00 ± 0^a^
60	50.00 ± 0^c^	72.50 ± 2.04^b^	96.67 ± 4.71^a^
80	54.17 ± 1.17^bc^	70.00 ± 5.07^b^	100.00 ± 0^a^
100	55.00 ± 0^bc^	65.00 ± 5.80^b^	100.00 ± 0^a^
121	65.00 ± 7.36^a^	97.50 ± 3.53^a^	99.17 ± 1.18^a^
F-value	5.351^*^	8.917^**^	0.882
pH			
2	85.00 ± 2.02^a^	77.50 ± 2.04^bc^	100.00 ± 0^a^
4	79.17 ± 1.17^b^	70.83 ± 3.12^c^	100.00 ± 0^a^
6	75.00 ± 0^bc^	90.00 ± 0^a^	100.00 ± 0^a^
8	72.50 ± 2.00^c^	82.50 ± 5.40^ab^	98.33 ± 2.36^a^
10	70.83 ± 3.11^c^	89.17 ± 3.11^a^	96.67 ± 3.12^a^
12	72.50 ± 2.01^c^	82.50 ± 4.00^ab^	100.00 ± 0^a^
F-value	14.541^***^	9.012^**^	1.527

*COS* chitooligosaccharides

Results are expressed as mean ± standard deviations of values from triplicate experiments

^*^Significant difference at *p* < 0.05

^**^Significant difference at *p* < 0.01

^***^Significant difference at *p* < 0.001

The values in the same column with the same letter are not significantly different

The EI_24_ of the aniline COS solution showed little fluctuation and was almost constant from 97 to 100%, when the temperature was changed from 40 to 121 °C. While the emulsification increased at 121 °C, the emulsification indices of the motor oil and burned motor oil were both thermally stable and showed a slight reduction with temperature increases. Regarding the change in pH, the emulsification of the aniline (96.67–100%) held up well between pH 2.0 and pH 12.0.

The emulsification of motor oil by COS slightly decreased with the pH increase, especially at pH 8.0, for which an emulsification index of 70.83% was obtained, while emulsification indexes of 70.83–90.00% were obtained with burned motor oil in the pH range of 2–12.

#### Dispersion capacity

The process of dispersing the hydrocarbon into tiny emulsions into the aqueous phase is known as dispersion. An initial experiment was conducted to confirm the removal of hydrocarbons from motor oil-contaminated water and burned motor oil in order to assess the effectiveness of COS as a dispersant. For both oils, the COS displayed dispersion activity (Data not shown).

#### Hydrocarbon removal from contaminated sand

Following treatment with COS, SDS, or distilled water (used as a control), the percentage of hydrocarbon removal (motor oil, burned motor oil, and aniline) from the contaminated sand was reported in Fig. 5B. The results demonstrated that adding the biological COS (emulsifier) promoted an improvement in the elimination of hydrocarbons. Distilled water was only able to remove 1.20% of the motor oil that was contained in the sand. Chemical surfactants (SDS) demonstrated the ability to remove hydrocarbons, and these values were almost identical to those found by the COS, demonstrating the bioproduct’s efficacy.

#### Infrared spectroscopy of the residual hydrocarbons

The FT-IR spectrum analysis verified the presence of several functional groups in the remaining hydrocarbons. Every hydrocarbon is made up of various functional groups that are associated with aromatic, aliphatic, and alkane hydrocarbons. A peak was seen at 3396 cm^−1^ in motor oil treated with H_2_O (Fig. 5C).

Similar bands to those discovered in motor oil were present in the burned motor oil. Due to compound degradation and by-product generation, some bands in burned motor oil treated with COS got stronger while some bands showed decreased intensity (Fig. 5D).

Figure 5E displays the aniline and its degradable product’s FTIR spectra. Specific bands correspond to 3260 cm^−1^ wave numbers in infrared transmittance of aniline. This band is positioned lower than the O–H vibration stretching band, which is almost at the same wave number as aniline treated with COS (3287 cm^−1^).

## Discussion

Petroleum hydrocarbons are a major environmental risk due to their toxicity. Petroleum wastes cannot be effectively removed by conventional procedures, which are expensive. Consequently, it’s necessary to look for safe and affordable methods for bioremediating these hydrocarbons. Products that have broken down from chitin, like COS, provide an innovative safe way to remove hydrocarbons. We needed to produce chitin-degrading enzymes effectively to achieve a high level of COS generation. The first major investigation in this study focused on optimizing the production of chitinase A. The second investigation involved the application and evaluation of chitooligosaccharides, which are released from chitin biodegradation by chitinase A, as a bio-emulsifier factor. For the first investigation two approaches were performed, PBD and RSM statistical optimization were conducted to optimize the native chitinase production from the wild strain *S. marcescens* NRC408. The second approach  involves cloning and expressing the *SmChiA* gene from *S. marcescens* NRC408 in an expression host *E. coli*.

For the first approach, seven variables were examined for chitinase production using PBD. The main effect of the variables revealed that shrimp waste, raffinose, wheat bran, and KH_2_PO_4_ had a negative main effect, whereas corn steep liquor, time, and inoculum had a positive main effect. The production of chitinase enzymes is affected by many factors, such as the carbon source and the nitrogen source like wheat bran and corn steep liquor [20]. Also, inoculum size and time affected chitinase production [21]. The highest activity obtained by PBD was 41.12 U/mL. In addition, the P-values showed that the most important factors influencing the production of chitinase A were raffinose, wheat bran, and corn steep liquor, then RSM was used to determine the levels of essential factors for the maximum yield of chitinase [22]. The RSM increased the chitinase activity to 78.56 U/mL, which was estimated to be 3.56 times higher than the basal medium activity, which was greater than the prior report using the RSM approach and raised by almost 2.1 times [22].

The second approach used to improve chitinase production is isolation, cloning and heterologous expression of *SmChiA* gene. The open reading frame of *SmChiA* gene was 1692 bp; Tao et al. [23] reported a similar result. The *SmChiA* gene encodes 563 amino acids with a signal peptide consisting of 23 amino acids as reported by Wu et al. [24]. Different IPTG concentrations and incubation temperatures were used to promote the production of recombinant chitinase A in *E. coli*; the ideal IPTG concentration was 0.2 mM, and the ideal temperature was 37ºC. By optimizing the temperature and IPTG concentration, the production of recombinant proteins increases as indicated by Sadeghi et al. [25]. When higher concentrations were used, the proteins were visible but inactive, indicating the formation of inclusion bodies. Previous studies by Yan et al. [26] and Ariyaei et al. [27] have also examined this topic. The purified recombinant chitinase was analyzed by SDS which revealed a band with a molecular weight of 58 kDa corresponding to chitinase A protein that was found to be secreted by *S. marcescens* [28], although 57 kDa of chitinase A was reported by Danismazoglu et al. [29]. The activity of the purified recombinant chitinase is 228.085 U/mL, which is 2.9 times higher than the activity obtained using the statistical optimization technique for the wild type strain. Also, Okay and Alshehri reported that the chitinase gene from *S. marcescens* was successfully cloned and overexpressed in *B. subtilis* 168. The recombinant chitinase was 2.15 times more than the parental strain [30]. Various factors, such as the methods of protein synthesis, cell membrane viscosity and permeability, normal cell physiology, energy metabolism, and oxygen uptake, can potentially influence the production of enzymes during the fermentation process [22]. In addition to chitinase A, several other enzymes are produced during the fermentation process of *S. marcescens* NRC408 on shrimp powder. These enzymes may impact the role of chitinase in the chitin biodegradation process. Heterologous expression is one way to overcome this obstacle and reduce the bottlenecks associated with intrinsic host expression. However, this method presents a challenge in terms of matching suitable expression elements with specific expression hosts and improving the conditions for induction of expression [31]. In our study, the *E. coli* strain BL21 (DE3) expression system and pET28a (+) have shown their effectiveness in producing functional and high-yielding chitinase A. This expression strategy has been successfully employed in previous research to produce soluble and functional recombinant chitinase in a heterologous manner [29]. When considering the production and utilization of chitinase A from an economic standpoint, it becomes evident that establishing an in vivo factory connected to the transformant expression host (*E. coli* (DE3) and employing these enzymes in a purified form through ethanol precipitation and DEAE-Sephadex ion exchange column represents an exceptionally cost-effective approach. This stands in stark contrast to the expenses involved in chemical methods.

Since the recombinant chitinase exhibits activity higher than that obtained by optimizing the production conditions from *S. marcescens* NRC408, the recombinant chitinase was chosen for further studies. The biochemical analysis of the recombinant chitinase A reveals that pH 5.5 was determined to be the optimal pH. This finding was more in line with Emruzi et al. [32] at pH 5.0, which has the maximum chitinase activity for *S. marcescens*. Nonetheless, the optimal pH of *S. marcescens’* recombinant chitinase A is 9.0 [30]. Furthermore, the recombinant chitinase A optimal temperature was 65 °C, which is higher than the previously reported, 60 °C [30, 32], and at 45 °C [33] for chitinase activity. It is advised to use a high temperature for biotechnological applications. Furthermore, the activation energy of chitinase A was found to be 4.202 kJmol^−1^, which is closer to Preety and Hooda’s value [34].

 For the second investigation, research was done on recombinant chitinase A capacity to hydrolyze shrimp waste. The outcomes demonstrated that chitinase A could hydrolyze autoclaved shrimp shells quickly, releasing a significant quantity of GlcNAc-reducing sugar in the process. This suggests that the enzyme that was obtained was an exochitinase because of its capacity to hydrolyze GlcNAc. Chitinase A was first categorized as a *N*-acetyl-glucosaminidase, which is consistent with the findings of Wang et al. [35]. Furthermore, an estimation of the COS level produced was made. Additionally, the ^1^H NMR spectra provided more insight into the molecular structures of COS. The presence of GlcNAc residue was confirmed by COS, which displayed a characteristic signal peak at δ 1.92 ppm, which corresponded to the methyl protons of the N-acetyl groups [36]. The COS backbone’s proton signals were found at 2.92 ppm (H2) and 3.28–4.28 ppm (H3–H6) [37, 38]. According to Kim et al. [39], the protons in the glucopyranosyl ring were situated in the range of 3.3–4.6 ppm.

One sensitive and specific analytical technique for finding COS is LC/MS spectroscopy [40]. The recombinant chitinase A enzyme produced COS, and its ESI/MS spectra were acquired in the positive mode. Because of evidence in the literature that GlcN, GlcNAc, and COS easily produce protonated adducts in ESI, this technique has been widely used in the positive ionization mode [40–42]. [M + H]^+^ ion peaks make up all of the peaks. Six major peaks were identified, two of which were monomers, and the others were COS. The mass value of 268.12 (Rt; 1.01) for the monomer can be ascribed to *N*,*N*′-diacetyl d-glucosamine. An ion with the same monomers is represented by the signal at 268.12 m/z [43].

An evaluation of COS’s effectiveness as a bioemulsifier revealed that it could emulsify a variety of hydrocarbons, confirming its suitability for hydrocarbon-polluted media bioremediation by dispersing and increasing the availability of the hydrocarbons that were recalcitrant [44]. One of the most crucial aspects to consider is the capacity to establish and stabilize emulsions. Since biosurfactant/emulsifier (COS) are substrate-specific, their capacity to emulsify hydrocarbons depends on the hydrophobic compound. The compatibility between the hydrocarbon and the conformational structure of the biosurfactant determines the emulsification capacity and determines whether or not the microscopic droplets can be stabilized [45]. The emulsification performance of COS is significantly influenced by its molecular structure, molecular weight, and functional groups. The significant emulsification ability of exopolysaccharide can be attributed to the combination of electrostatic contact and hydrophilic groups [46].

To the author’s knowledge, there is no reports on COS produced from engineered chitinase enzyme as an emulsifier. COS containing many active groups, such as –NH_2_ and –OH, and a small amount of *N*-ethylphthalide amino group. Their good biocompatibility and biodegradability are therefore the reason for their widespread application in food and beverage research as well as related fields. Through interaction with surface-active agents, adsorption of the protective layer at oil–water interfaces, and viscosity improvement, they can function as emulsifiers and emulsion stabilizers. 

Investigations were conducted on salinity, temperature, and pH as well as other parameters that affect COS’s efficacy. The ability of emulsifying agents to preserve the stability of substances with varying degrees of polarity—which are reflected by EI_24_ above 50% after 24 h [47]. The results indicate that the COS’s durable properties are highly advantageous for environments with harsh requirements, like oil recovery and the bioremediation of contaminated marine environments.

The efficiency of the COS for the removal of hydrocarbons from water contaminated with motor oil and burned motor oil was examined. The outcomes verified that the COS addition facilitated an improvement in hydrocarbon removal. Targeting oily areas of the ocean may be made easier by the COS’s high dispersing activity for both oils. Surfactants, which are in charge of solubilization and dispersion, make up the majority of an oil dispersant’s composition. Surfactants’ amphipathic nature allows for the creation of micelles, which disperse in water to solubilize oil [45]. Additionally, the effectiveness of COS in eliminating hydrocarbons from polluted sand was investigated. The findings demonstrated how important the COS addition was to the cleaning procedure. Biosurfactants have the ability to emulsify hydrocarbons, improving their water solubility, lowering surface tension, and promoting the displacement of oil compounds from soil particles [48, 49]. Costa et al. [50] suggest that through certain interactions, biosurfactants can also encourage the movement of hydrophobic pollutants towards the aqueous phase, leading to emulsification and micellization, which makes it easier for the contaminants to be removed or biodegraded. It is crucial to note, however, that despite their comparable effectiveness in this application, chemical surfactants are poisonous and have limited biodegradability, which makes them detrimental to ecosystems [51]. COS may be used for increased oil recovery and contaminants cleansing. Because of this feature, it is a prospective COS for the advancement of technical procedures utilized in the bioremediation of hydrocarbon-contaminated locations.

The FT-IR spectrum analysis verified the presence of several functional groups in the residual hydrocarbons. Every hydrocarbon is made up of various functional groups that are associated with aromatic, aliphatic, and alkane hydrocarbons. A peak at 3396 cm^−1^, corresponding to the –OH stretching bands, was seen in motor oil treated with H_2_O [52]. The CH valence vibrations of saturated n-alkyl groups are represented by the spectral bands with peaks at 2953, 2920, and 2853 cm^−1^. The CH_2_ and CH_3_ groups are represented by symmetric and asymmetric deformation vibrations of CH bonds that show a spectrum with peaks at 1458 cm^−1^ and 1377 cm^−1^ [53]. The location of P-O-C bonds is 1090–920 cm^−1^ [54]. The aromatic content’s spectrum characteristics at approximately 800 cm^−1^ (874 cm^−1^) are investigated [55]. The vibrations of the CH bonds in the long carbon chains are represented by a frequency in the spectrum of 721 cm^−1^. Peaks at 3396, 1620, 1026, and 874 cm^−1^ disappeared in the comparison, demonstrating an effective degradation by COS. Furthermore, two additional peaks were formed at 1512 and 604 cm^−1^.

There were bands identical to those found in motor oil in the case of burned motor oil. The hydroxyl (–OH) group is responsible for the broad feature that is seen in IR spectra, which is centered on 3374 cm^−1^ and represents water [55]. Due to compound decomposition and by-product generation, some bands in burned motor oil treated with COS became stronger while some bands showed reduced intensity. Carboxylic acid (C–O) was detected at 1030 cm^−1^. Burned motor oil treated with COS (1026 cm^−1^) had significantly more C–O than burned motor oil treated with water (1030 cm^−1^). Likewise, the C–C or C–H stretch duplets detected in the degraded test by COS at 1456 and 1377 cm^−1^ are significantly more pronounced than the corresponding duplets detected in the samples treated with water at 1459 and 1376 cm^−1^. When the aliphatic and aromatic components of the crude oil mineralize during degradation, alcohols and acids are formed. This may be the cause of the peak that is seen in the burned motor oil that has been degraded by COS and is not in the control sample. This peak indicates the –OH stretching bands [55].

Secondary amine stretching (N–H) vibrations are represented by the bands in the 3000–3600 cm^–1^ range [56]. At wave numbers of 3260 cm^−1^, aniline exhibits unique bands for Infra-Red transmittance. The band of vibration stretching of O–H appears lower than that of aniline treated with COS, virtually at the same wave number (3287 cm^−1^). The stretching frequencies which correspond to particular functional groups in the spectra of aniline treated with water are 2921 cm^−1^ (aliphatic C–H stretching) [57], 2851 cm^−1^ (symmetric and asymmetric stretching vibration of C–H) [58], 1576 cm^−1^ (aromatic –C–H stretching symmetric and asymmetric), and 1342 cm^−1^ (asymmetric S–O stretching vibrations) [59]. The aromatic ring’s C=C stretching and the secondary aromatic amine’s C–N stretching are responsible for the main absorbances that are shown at 1443 cm^−1^ and 1295 cm^−1^ [60, 61]. The aromatic hydrocarbon C=CH stretching vibration may be connected to the band at 600 cm^−1^ [62, 63]. There are stretching vibrations of the benzene C–H in the 3100–3000 cm^−1^ range [63]. The C–H stretching of aniline treated with H_2_O was at 3057 cm^−1^ and not detected for aniline treated with COS. The peaks at 1576 cm^−1^ and 1502 cm^−1^ were observed only in aniline treated with H_2_O. The removal of these bonds from aniline with COS biodegradation suggests that these compounds were produced from aniline and progressively broke down into various intermediate products. This occurrence proved that the COS could biodegrade a variety of hydrocarbon molecules.

## Conclusion

This study provides an effective improvement for the productivity of a novel chitinase from *S. marcescens* NRC408. The production of chitinase A from *S. marcescens* NRC408 was improved using two approaches, statistical optimization and heterologous expression system, the latter one shows overexpression of chitinase A with an estimated activity of 228.085 U/mL, representing a 2.9-fold increase in activity compared with the statistical optimization method. The purified recombinant chitinase A was used to hydrolyze shrimp shells to produce a high level of COS which was evaluated as an effective bioemulsifier. Furthermore, it produced stable emulsions under harsh pH, salinity, and temperature conditions for an extended period. Since COS is a unique microbial emulsifier that has been carefully designed by methods to have great potential for green technology, its stability is a crucial factor in determining its implementation in biotechnological applications in agriculture and bioremediation. The FTIR data demonstrated how effectively the COS contributed to the hydrocarbons’ breakdown. Because of its affordability and environmental friendliness, COS could therefore be used to improve the biodegradation of complex hydrocarbon components, including marine oil spills.

## Materials and methods

### Bacterial strains and plasmids

The chitinase enzyme was sourced from the bacterial strain *S. marcescens* NRC408. This bacterium was genetically identified through sequencing the 16S rRNA bar-coding gene, resulting in the assignment of accession no. OR793165 in GenBank. It was graciously obtained from the microbial deposit at the Department of Microbial Genetics, National Research Centre, Egypt.

*E. coli* DH5α used as a host for gene cloning, and *E. coli* BL21 (DE3) for gene heterologous expression. The Lysogeny broth (LB) media were used for bacterial cultivation. Gene cloning was performed by insertion of the gene fragment into CloneJET PCR Cloning Kit (Thermo Scientific™ Waltham, MA, USA). pET-28a (+) vector from Novagen was used for gene expression.

### Chitinase production and enzyme activity estimation

In order to evaluate chitinase production in *S. marcescens*, specific body parts of shrimp were purified to create shrimp powder, serving as a chitin source. The heads, shells, and tails were meticulously cleaned, then dried in an oven at 60 °C until suitable for grinding and sieving into fine powder. In a 250 mL Erlenmeyer flask, the following components (g/L) were added: shrimp shells (40 g), corn steep liquor (15), wheat bran (10), raffinose (2), and KH_2_PO_4_ (10). The volume was adjusted to 50 mL and autoclaved. After cooling under aseptic conditions, 2.5 mL of the overnight bacterial culture of *S. marcescens* was inoculated and kept at 30 °C for 48 h under agitation at 180 rpm. The culture was centrifuged at 12,000 rpm for 20 min, and the resulting supernatant served as the enzyme source. For the substrate, 0.1% (w/v) of the synthetic substrate 4-Nitrophenyl *N*-acetyl-β-d-glucosamine was dissolved in 0.05 M acetate buffer at pH 4.5, following the procedure outlined by Rustiguel et al. [64]. Exo-chitinase activity was determined by the release of *p*-nitrophenol, indicated by a yellow color change. Absorbance was measured at 410 nm using a spectrometer. One unit of the enzyme corresponded to the release of 1 μmol of *p*-nitrophenol per minute. Protein content in the clear supernatant was quantified by Lowery method [65].

### Statistical optimization of chitinase A production

There were two stages to the optimization study. In the first, Plackett Burman Design (PBD) was used to determine the main parameters that effectively affect the production of chitinase A [66]. In the second step, response surface methodology (RSM) of central composite design (CCD) was used to estimate the optimal chitinase A synthesis. The optimal point was reached by identifying which three factors significantly increase the production of chitinase A [10, 67].

### Plackett–Burman experimental design

Through assessing seven independent factors (shrimp waste, raffinose, wheat bran, KH_2_PO_4_, corn steep liquor, time, and inoculum) in nine combinations organized by the PBD matrix [66], *S. marcescens* 408 produced chitinase A during submerged fermentation. A high (+) and low (−) level was assessed for every trait (Table 1A). PBD was established using the first-order linear model (Eq. 3):

3 $${\text{Y}} = {\text{ B}}0 + \Sigma {\text{ BiXi}},$$

where Y is the response (chitinase A production), Xi is the level of the independent variable, B0 is the model intercept, and Bi is the linear coefficient. Each variable’s principal effect was determined using Eq. (4):

4 $${\text{E}}\left( {{\text{Xi}}} \right) = { 2}(\Sigma {\text{ Mi}} + - {\text{Mi}} - )/{\text{N}},$$

where the influence of the examined variable was represented by E(Xi). The variables Mi + and Mi − , which denote the number of trials (N) and the high and low levels of the independent variable (Xi) assessed in those trials, were used to represent chitinase A production. The significance level, also known as the p-value, for each investigated variable was determined using the student’s t-test.

### Central composite design

Three variables (wheat bran, raffinose, and corn steep liquor) were selected for the RSM of CCD. The experimental design included 13 runs (Table 2), and the independent variables were investigated at five different levels. To fit the second-order polynomial function in Eq. (5), it was determined how the independent variables and the anticipated response, Y, related to one another.

5 $$Y_{Activity} = {\beta}0 + \, {\beta}1X1 + \, {\beta}2X2 + \, {\beta}3X3 + \, {\beta}11X12 + \, {\beta}22X22 + \, {\beta}33X32 + \, {\beta}12X1X2 + \, {\beta}13X1X3 + \, {\beta}23X2X3,$$

Y_Activity_, the predicted chitinase A production (U/mL), has the following coefficients: linear (β1, β2, and β3), cross-product (β12, β13, and β23), quadratic (β11, β22, and β33), and independent variables (corn steep liquor, raffinose, and wheat bran, respectively). Regression analysis was performed on the collected experimental data using "SPSS" Version 15.0. The degree to which the polynomial model equation matches the data was expressed as a coefficient of determination, or R^2^. The tests were conducted in three duplicates, and the mean values were recorded.

### Gene cloning and heterologous expression of Chitinase encoding gene (*SmChiA*)

#### Primer design, DNA isolation, and *SmChiA* gene amplification

The design of primers for the amplification of the *SmChiA* gene was based on the analysis of the chitinase A protein sequence from the Uniprot database (https://www.uniprot.org/uniprotkb/P07254/entry) and the *ChiA* gene sequence obtained from GenBank (https://www.ncbi.nlm.nih.gov/nuccore/L01455) (accession number L01455.1). Primer design was executed using the Primer3 program (https://bioinfo.ut.ee/primer3-0.4.0/). The forward primer, SmChiA-F (5′-GCGAATTCATGTCCACACGCAAAGCCGTTA-3′), includes an *EcoRI* recognition site (underlined), while the reverse primer, SmchiA-R (5′-AAGCTTTTATTGAACGCCGGCGCT-3′), contains a *HindIII* recognition site (underlined). Restriction enzymes were provided by New England Biolabs (NEB), and the primers were synthesized by Macrogen in Korea. PCR amplification of the *SmChiA* gene was performed using Phusion High-Fidelity DNA Polymerase from Thermo Scientific™ according to the manufacturer’s protocol, with an annealing temperature of 60 °C.

#### Cloning and transformation of *SmChiA* gene

The PCR product was resolved on a 1.2% agarose gel. The band comprising DNA fragment was cut out and processed using GeneJET Gel Extraction Thermo Scientific™ (Waltham, MA, USA). The isolated *SmChiA* gene fragment including the ORF was inserted into pJET1.2/blunt using the CloneJET PCR Cloning Kit Thermo Scientific™ (Waltham, MA, USA), according to the instructions. Preparation of *E. coli* DH5α competent cells was carried out by Sambrook and Russell method [68], which were then transformed with the ligation product. To select successful transformants, cells were grown on LB agar plates with a carbenicillin concentration of 50 µg/mL for 18 h at 37 °C. Positive transformants were identified by the presence of developing colonies, as the vector contains a lethal restriction enzyme gene that is destroyed when a DNA insert is ligated into the cloning site, allowing only bacterial cells carrying recombinant plasmids to form colonies. The resulting vector was termed pJET-SmChiA.

#### Heterologous expression of chitinase A in *E. coli* BL21 (DE3)

The GeneJET Plasmid Miniprep Kit from Thermo Scientific™ (Waltham, MA, USA) was used to extract the modified vector, pJET- SmChiA, from transformed cells as per the instructions. The pJET-SmChiA and pET-28a (+) vectors were then digested simultaneously with *EcoR1* and *HindIII*. Following digestion, the resulting products were resolved using 1.2% agarose gel. The GeneJET Gel Extraction Kit (Thermo Scientific™) was used to isolate two bands: the gene fragment and pET-28a (+). These bands were then ligated using T4 DNA Ligase (Thermo Scientific™) following the indicated manufacture instructions. The ligated product was ready for introduction into competent *E. coli* DH5α cells, which were then grown under kanamycin selection (50 µg/mL in LB agar). All gene cloning and transformation steps followed Sambrook and Russell’s techniques [68]. Transformant cells that developed under kanamycin selection were selected and propagated in LB broth containing 50 µg/mL kanamycin. The positive transformant containing SmChiA-pET28a clones and the integrity of the recombinant plasmid were verified using two methods: colony PCR with a set of primers for the target gene and pET-28a (+) T7 promoter primers, and double digestion of the recombinant vector with *EcoRI* and *HindIII*. Following validation of the recombinant vector’s proper structure, it was extracted and introduced into *E. coli* BL21 (DE3) competent cells, which were grown under kanamycin selection.

#### Expression of chitinase A in *E. Coli* BL21 (DE3), optimization, purification and SDS-PAGE

The positive transformant containing the pET- SmChiA construct was grown on LB agar substrate with kanamycin (50 µg/mL). A single colony from a clone was used to inoculate 5 mL of LB broth with kanamycin (50 µg/mL), then incubated at 37 °C in a shaking incubator. One milliliter of the overnight culture was used to inoculate 200 mL of LB broth with kanamycin (50 µg/mL) in a 500 mL culture flask. The culture was then incubated at 37 °C with vigorous shaking. After reaching a mid-log phase of 0.6 at OD600, isopropyl β-d-1-thiogalactoside (IPTG) was added at various doses (0.1, 0.2, and 1 mM) and incubation temperatures (18, 28, and 37 °C). The culture was shaken for an additional 16 h following induction [69]. The cells were then collected, washed in deionized water, and suspended in 10 mmol/L PBS at pH 7. The cells were sonicated for 10 min, with cycles of 30 s of sonication followed by 30 s of rest, while kept on ice. The resulting cell lysate was then centrifuged for ten minutes. Chitinase protein was precipitated using the previously reported techniques [70, 71]. In brief, a volume of roughly 250 mL of lysate was treated to fractional precipitation at 4 °C using cooled ethanol concentration at 50–60%. Then, DEAE-sephadex ion exchange column is used for chitinase purification [72]. The isolated target protein was analyzed using the Laemmli method [73] and 12% SDS-PAGE. Coomassie Brilliant Blue R-250 was employed for visual band detection, and a pre-stained protein ladder (Thermo Scientific™) assisted in determining the molecular weight of the chitinase A.

#### Sequencing of *SmChiA* gene, phylogeny and chitinase amino acid analysis

The vector primers for pET28a (+), namely pET-F (5′-CGTCCGGCGTAGAGGATC-3′) and pET-R (5′-ATCCGGATATAGTTCCTCCTTTC-3′), were used to determine the nucleotide sequence through the dideoxynucleoside chain termination method [74] using Big Dye terminator cycle sequencing ready reaction kits in biomedical laboratory of colors (Clinilab, Egypt). After sequencing, sequence mistakes were corrected using editing, and unreadable parts at the 3′ and 5′ ends were removed with trimming. These processes were carried out using the BioEdit software version 7.0.2. The modified sequence was aligned using the NCBI nucleotide database. (https://blast.ncbi.nlm.nih.gov). The N-terminal signal peptide was analyzed using the SignalP version 6.0 program (http://www.cbs.dtu.dk/services/SignalP/ [75]. The molecular mass and isoelectric point (pI) of the encoded chitinase A were determined using the ExPASy website (http://www.expasy.ch/tools/protparam.html. MEGA11 was used to determine the evolutionary relationship between chitinase A’s deduced amino acid and other proteins with high identity [76]. The MUSCLE algorithm [77] was used to align multiple sequences. The evolutionary history was determined using a 1000 repetitions bootstrap test [78], and the neighbor-joining method [79] was used to infer the evolutionary history.

### Biochemical characterization of recombinant chitinase A

#### Effects of pH and temperature on enzyme activities

Acetate and phosphate buffer solutions were utilized to measure the activity of the recombinant chitinase A under various pH ranges (3.5–7). The pH stability of the enzyme was then examined without a substrate by letting it sit at room temperature in the aforementioned buffer for varied time. Eventually, under standard test conditions, the residual enzyme activity was computed.

The optimal temperature for chitinase A was determined by incubating the enzyme and pNPg combinations for 15 min at various temperatures (ranging from 25 to 70 °C). The activation energy (Ea) of the produced enzyme was calculated using the Arrhenius plot (ln relative activity vs. reciprocal of temperature in Kelvin), as shown in the following equation:

6 $${\text{Slope}} = - {\text{Ea}}/{\text{R}},$$

where R represents the gas constant. In the meantime, the thermal stability of chitinase A was investigated using the remaining activity of enzyme solutions that had been pretreated for 30 min at various temperatures.

#### Hydrolysis of shrimp powder by recombinant chitinase

Twenty mg of previously produced shrimp powder were mixed in 1 mL of 0.05 M phosphate buffer (pH 5.5) and subjected to three distinct treatments: boiling for 10 min, microwaving for 1 min and autoclaving for 16 min. After adding 1 mL of chitinase A (5U) to the mixture at 45 °C, the sample was hydrolyzed for 1, 2, 3, 4, 5, 6, 7, and 8 h in an electric water bath kept at a constant temperature. The enzyme was then deactivated by heating it to 100 °C for 10 min. The hydrolyzate was centrifuged for 10 min at 4000 rpm, and the amount of reducing sugar that was released throughout the process was calculated from the supernatant.

#### Estimation of oligosaccharide level

The level of released reducing sugars was used to calculate the total amount of COS that was released [80]. The hydrolysis percentage is calculated by total reducing sugars content in the cell-free supernatant (mg/g dry shrimp)/total carbohydrate content of complete acid hydrolyzed SPB (mg/g dry shrimp) × 100. The total carbohydrate content was determined for shrimp [81] after its complete acid hydrolysis [82].

### Characterization of COS

#### Nuclear magnetic resonance

The ^1^H-NMR spectra of COS were obtained using D_2_O as the solvent in a BRUKER spectrometer running at 400.15 MHz. Parts per million (ppm) chemical shifts were reported on the δ scale, and spectra were collected at 300 K.

#### Mass spectrometry (LC–MS)

Liquid chromatography linked to mass spectroscopy was used to separate and identify COS. The used mass spectrometer was an XEVO TQD triple quadruple instrument from Waters Corporation, Milford, MA01757 USA. The ACQUITY UPLC–BEH C 18–1.7 μm to 2.1 × 50 mm C 18 column was used for the separation. There was a 0.2 mL/min LC flow rate. Eluent A (water + 0.1% formic acid) and Eluent B (acetonitrile + 0.1% formic acid) were applied in a multi-step linear gradient. A COS solution of 10 μL was injected. The solvent of choice for the analysis was pure H_2_O. The Waters mass spectrometer was connected to the LC system via an ambient pressure electroscopy link. In positive ionization mode, the ESI source was set.

### Evaluation of chitooligosaccharides (COS) as an emulsifier and its application in hydrocarbon removal

#### Emulsification index

The emulsification index was used to assess the COS’s capacity to emulsify hydrocarbons, including vegetable oil, motor oil, motor burning oil, benzene, toluene, xylene, petroleum ether, cyclohexane, and aniline. For this purpose, equal amounts of the hydrocarbons and an aqueous COS (5, 10, 20 mg/mL) solution were combined, vortexed for 2 min, and then allowed to stand for 24 h. The height of the emulsion layer divided by the overall height of the liquid column yields the percentage of the emulsification index (E_24_) [83]. Assays were performed in triplicate.

#### Stability of COS to environmental stress

The stability of COS (20 mg/mL) was studied under a wide range of temperatures, pH, and different salt concentrations. A wide range of temperatures, pH values, and salt concentrations were investigated in order to examine the stability of COS (20 mg/mL). The effect of the addition of different concentrations of NaCl on the activity of the COS was investigated. After adding a particular concentration of NaCl (0–12%, w/v), the emulsification activity was ascertained as mentioned earlier. The COS suspension was also kept at a steady temperature (40, 60, 80, 100, and 121 °C) for 30 min. After using 6.0 M NaOH or HCl to change the broth’s pH to 2, 4, 6, 8, 10, and 12, the impact of pH on emulsification was assessed.

#### Evaluation of the COS as a dispersant

The efficacy of COS as a dispersant was assessed using a visual test [84]. After slowly swirling with a slow magnetic stirrer, samples of motor oil and burned motor oil (5, 10, 20, 40, 80, and 100 μL) were carefully added to the water (20 mL) in a beaker until it was 1 cm deep. The COS was then added in an amount of 5.0 μL to the center of the vortex and agitated for 1.0 min at a maximum speed of 2000 rpm.

#### Hydrocarbon removal from contaminated sand

The bioemulsifier’s suitability for eliminating burned motor oil, motor oil, and aniline was examined [85]. In 250 mL Erlenmeyer flasks, 20 g of sand were put through the following treatments: (A) addition of 150 mL of distilled water (control), (B) addition of 150 mL of 0.5% SDS solution, and (C) addition of 150 mL of COS (20 mg/mL). The samples were deliberately contaminated with 20 mL each of motor oil, burnt motor oil, and aniline. The flasks were subjected to 150 rpm for 24 h at 30 °C and then centrifuged at 5000 rpm for 20 min for separation of the washing solution and sand sediment. The amount of oil remaining in the sand was gravimetrically determined by hexane.

#### FTIR analyses

As previously mentioned, an organic solvent was used to recover the residual hydrocarbons, which included motor oil, burned motor oil, and aniline. Fourier transform infrared (FTIR) analysis using an FTIR-800 spectrophotometer (Shimadzu, Japan) was performed on the organic solvent extract that contained the remaining hydrocarbons. For 100s, 64 scans of a wavelength between 400 and 4000 nm were conducted for the analysis.

#### Data analysis

Data processing was done using SPSS 17.0. To examine the variations between the samples, one-way ANOVA was employed. Duncan’s test was employed to illustrate the variations in individual means.

### Supplementary Information

**Additional file 1: Figure S1.** NMR **(A)** and MS analysis of chito-oligosaccharides obtained from enzymatic hydrolysis and illustrations of its m/z values (**B–G**).

## Data Availability

All data generated or analyzed during this study are included in this article.
