# Supplementary material for: Utilizing chitooligosaccharides from shrimp waste biodegradation via recombinant chitinase A: a promising approach for emulsifying hydrocarbon and bioremediation
Source: Microb Cell Fact. 2024 May 2;23:126. doi: 10.1186/s12934-024-02388-z (PMC11067288; doi:10.1186/s12934-024-02388-z)
Supplement: Supplementary file 1 — Additional file 1: Figure S1. NMR (A) and MS analysis of chito-oligosaccharides obtained from enzymatic hydrolysis and illustrations of its m/z values (B–G). [file 12934_2024_2388_MOESM1_ESM.docx]

**Additional information to the article “Utilizing of Chitooligosaccharides from Shrimp Waste Biodegradation via Recombinant Chitinase A: A Promising Approach for Emulsifying Hydrocarbon and Bioremediation” (Nour et al.)**

| **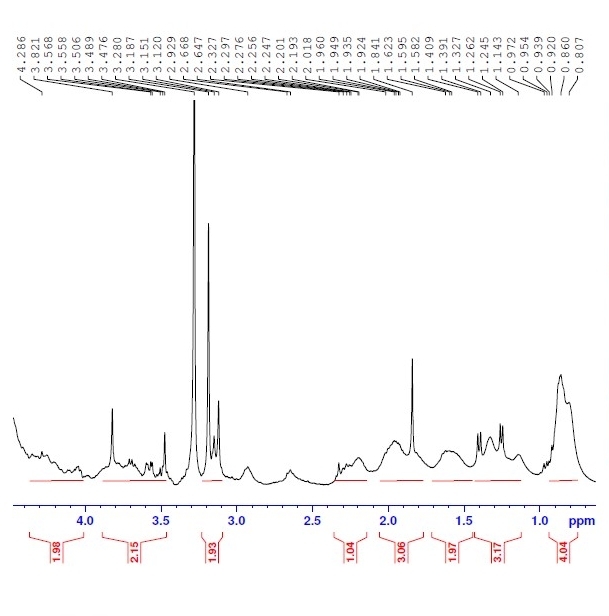(A)** |
| --- |
|  |
| **(B)** |
| **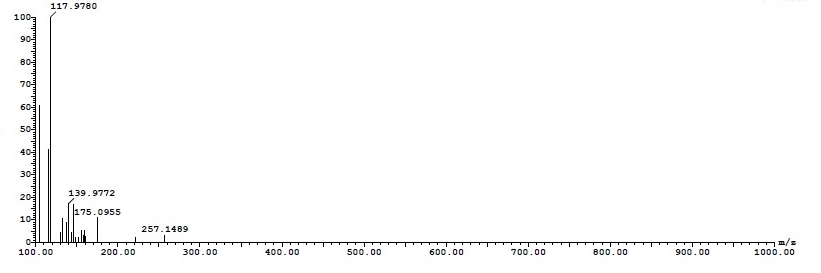** |
| **(C)** |
| **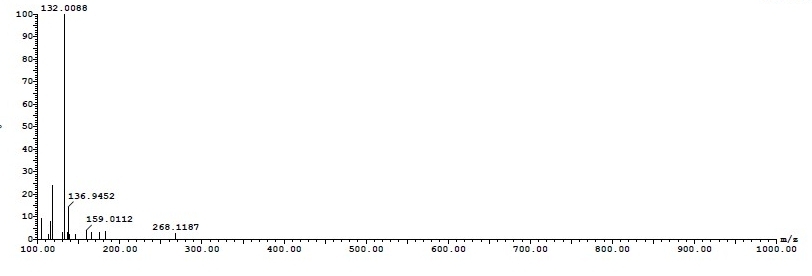** |
| **(D)** |
| **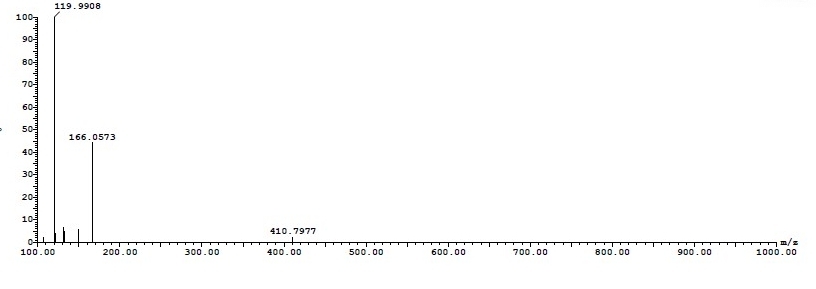** |
|  |
| **(E)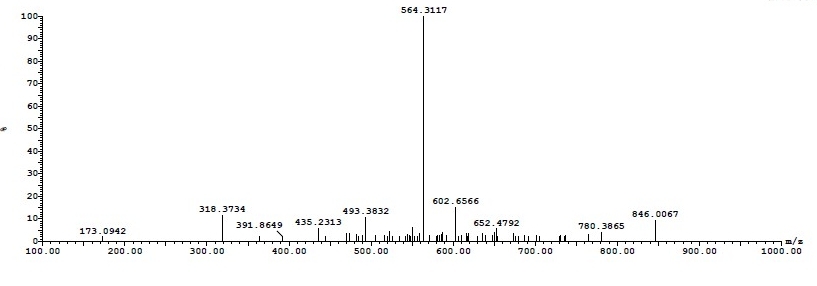** |
| **(F)** |
| **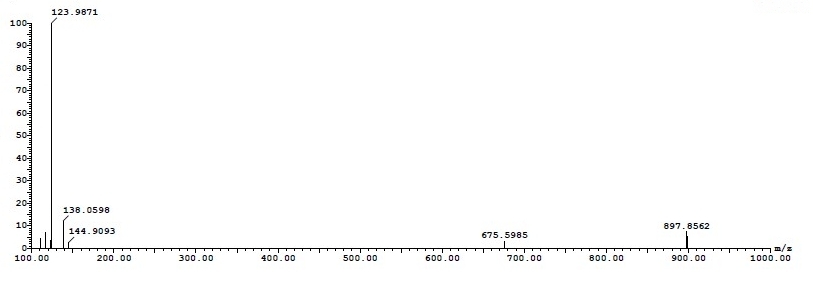** |
| **(G)** |
| **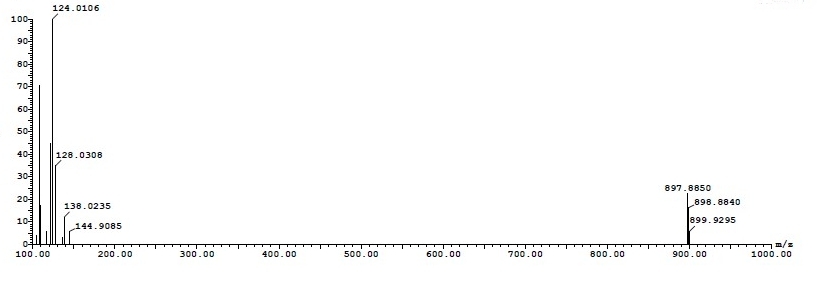** |

**Fig. S1**

NMR **(A)** and MS analysis of chito-oligosaccharides obtained from enzymatic hydrolysis and illustrations of its m/z values (B– G).
